# Supplementary material for: Characterizing Binding Interactions That Are Essential for Selective Transport through the Nuclear Pore Complex
Source: Int J Mol Sci. 2021 Oct 8;22(19):10898. doi: 10.3390/ijms221910898 (PMC8509584; doi:10.3390/ijms221910898)
Supplement: Supplementary file 1 [file ijms-22-10898-s001.zip › ijms-1381613-supplementary.pdf]

International Journal of
Molecular Sciences

Supplemental Information

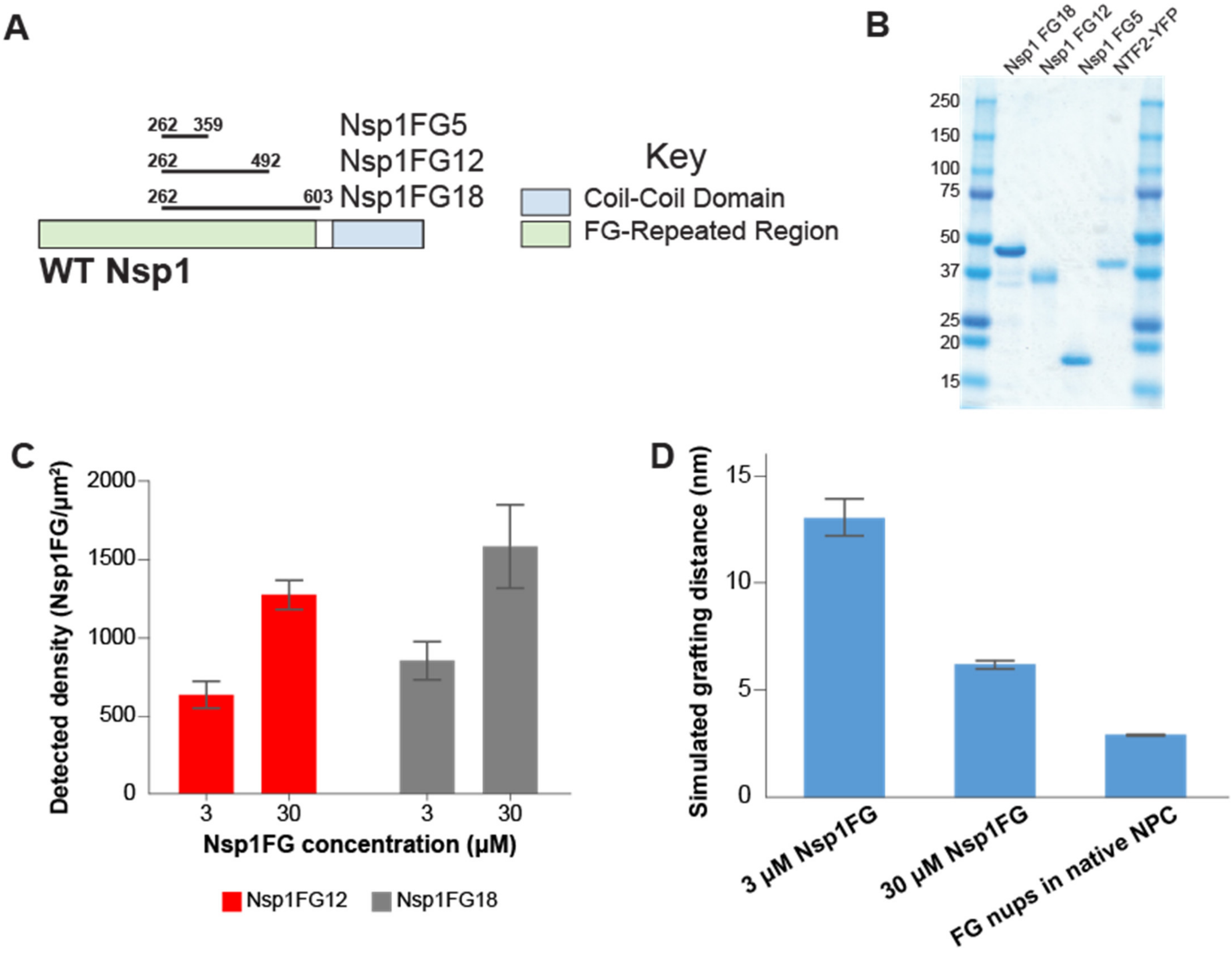

Figure S1. Average detected densities of Nsp1FG variants. (**A**) Scheme of Nsp1 fragments. (**B**) Nsp1 variants for surface attachment were highly purified, as confirmed with Coomassie blue stained SDS-PAGE gel. (**C**) Nsp1FG variants spanned the FxFG region of the WT Nsp1 FG Nup. (**C**) The average detected density of Nsp1FG12 and Nsp1FG18 surfaces. Error bars represent SEM. The Nsp1FG12 (3 µM) surface was assessed with 3 independent measurements, n=15 ROIs. The Nsp1FG12 (30 µM) surface was assessed with 4 independent measurements, n=20 ROIs. The Nsp1FG18 (3 µM) surface was assessed with 2 independent measurements, n=10 ROIs. The Nsp1FG18 (30 µM) surface was assessed with 2 independent measurements, n=10 ROIs. There is no difference between the Nsp1FG12 and Nsp1FG18 surfaces (3 µM or 30 µM, p-values 0.54 and 0.61 respectively) (**D**) Simulated grafting distances for Nsp1FG surfaces (based on 3 µM and 30 µM detected surface densities; n= 15 simulations) and for FG nups in native NPC. Native NPC grafting distance simulation was based on 160 proteins/NPC (with the surface area of the pore being roughly 0.01137 µm^2)[6, 22] or ~14000 proteins/µm^2 FG nup density within the pore; n=15 simulations. Error bars represent SEM.

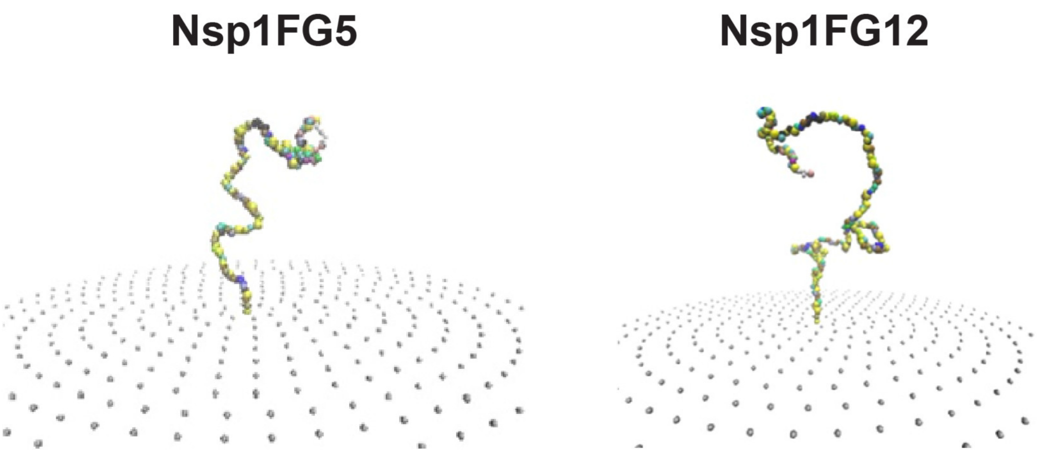

Figure S2. Snapshots of coarse-grained simulations of Nsp1 molecules.

A

	# of FGs	Charge pH 7.0	Molecular Weight (kDa)
Nsp1FG5	5 (1 FG, 4 FSFG)	3.6	15.6
Nsp1FG12	12 (1 FG, 11 FSFG)	6.6	29.5
Nsp1FG18	18 (3 FG, 15 FSFG)	3.7	41.1
Nsp1FG full domain (aa1-620)	30 (13 FG, 17 FSFG)	9.0	63.6

B

	30 μM Surfaces					3 μM Surfaces			
	NTF2 WT			NTF2 W7AI64A		NTF2 WT		NTF2 W7AI64A	
	K_d(nM)	$K_{d,10\%BSA}$(μM)	$K_{d,10\%\ YL}$(μM)	$K_{d,\ 10\%BSA}$(μM)	$K_{d,10\%\ YL}$(μM)	$K_{d,10\%BSA}$(μM)	$K_{d,10\%\ YL}$(μM)	$K_{d,10\%BSA}$(μM)	$K_{d,10\%\ YL}$(μM)
Nsp1FG5	200 ±21	15.3 ±1.2	8.7 ±2.7	3.2 ±0.8	No Binding	8.1 ±1.1	No Binding	1.1 ±0.6	No Binding
Nsp1FG12	400 ±35	4.4 ±0.4	2.0 ±0.5	7.8 ±0.6	No Binding	12.0±0.8	25.3 ±10.1	4.7 ±0.5	No Binding
Nsp1FG18	300 ±35	6.2 ±0.4	15.7 ±6.7	5.5 ±0.6	No Binding	6.5±0.8	26.4 ±9.0	No Binding	No Binding

C

	30 μM Surfaces					3 μM Surfaces			
	NTF2 WT			NTF2 W7AI64A		NTF2 WT		NTF2 W7AI64A	
	B_{max}	$B_{max\ (10\%\ BSA)}$	$B_{max\ (10\%\ YL)}$	$B_{max\ (10\%\ BSA)}$	$K_{d,10\%\ YL}$(μM)	$B_{max\ (10\%\ BSA)}$	$B_{max\ (10\%\ YL)}$	$B_{max\ (10\%\ BSA)}$	$B_{max\ (10\%\ YL)}$
Nsp1FG5	51.9 ±1.1	41.8 ±1.6	16.4 ±2.3	10.7 ±0.9	No Binding	24.7 ±1.3	No Binding	3.2 ±0.4	No Binding
Nsp1FG12	76.4 ±1.3	55.8 ±1.9	12.3 ±0.9	26.6 ±0.9	No Binding	48.5 ±1.5	19.3 ±4.3	23.6 ±0.9	No Binding
Nsp1FG18	74.7 ±2.0	57.0 ±1.5	24.9 ±5.4	29.5 ±1.2	No Binding	32.2 ±1.5	27.1 ±5.3	No Binding	No Binding

D

	K_d p-values		B_{max} p-values	
WT NTF2 FG5 vs WT NTF2 FG12	0.001665	*	1.24E-09	***
WT NTF2 FG5 vs WT NTF2 FG18	0.110682	ns	8.5E-08	***
WT NTF2 FG12 vs WT NTF2 FG18	0.086368	ns	0.482185	ns
WT NTF2 vs +BSA FG5	5.2E-09	***	0.000159	**
WT NTF2 vs +BSA FG12	3.06E-07	***	4.53E-07	***
WT NTF2 vs +BSA FG18	1.76E-09	***	5.01E-06	***
30 WT NTF2 +BSA vs Mut NTF2 +BSA FG5	9.51E-07	***	1.05E-10	***
30 WT NTF2 +BSA vs Mut NTF2 +BSA FG12	0.000568	**	1.42E-09	***
30 WT NTF2 +BSA vs Mut NTF2 +BSA FG18	0.320794	ns	0.020747	*
WT NTF2 +BSA 30 vsWT NTF2 +BSA 3 FG5	0.000748	**	2.94E-06	***
WT NTF2 +BSA 30 vsWT NTF2 +BSA 3 FG12	1.6E-06	***	0.010779	*
WT NTF2 +BSA 30 vsWT NTF2 +BSA 3 FG18	0.723771	ns	5.95E-08	***
3 WT NTF2 +BSA vs Mut NTF2 +BSA FG5	0.000158	**	2.22E-08	***
3 WT NTF2 +BSA vs Mut NTF2 +BSA FG12	1.08E-05	***	5.63E-08	***
3 WT NTF2 +BSA vs Mut NTF2 +BSA FG18	6.61E-06	***	8.02E-10	***
WT NTF2 +BSA vs WT NTF2 +YL FG5	1.53E-05	***	3.72E-09	***
WT NTF2 +BSA vs WT NTF2 +YL FG12	0.214234	ns	7.82E-05	***
WT NTF2 +BSA vs WT NTF2 +YL FG18	0.052834	*	0.368272	ns
30 WT NTF2 +BSA vs WT NTF2 +YL FG5	0.051027	ns	3.99E-06	***
30 WT NTF2 +BSA vs WT NTF2 +YL FG12	0.005167	*	1.62E-09	***
30 WT NTF2 +BSA vs WT NTF2 +YL FG18	0.186538	ns	0.000201	**
30 WT NTF2 +YL vs 3 WT NTF2 +YL FG5	0.013488	*	9.59E-05	***
30 WT NTF2 +YL vs 3 WT NTF2 +YL FG12	0.047697	*	0.146909	ns
30 WT NTF2 +YL vs 3 WT NTF2 +YL FG18	0.368034	ns	0.776100	ns

Table S1. NTF2-YFP Apparent K_d and Binding Capacity on Nsp1FG surfaces. (**A**) The properties of Nsp1FG variants and WT Nsp1 FG domain: numbers of FG domains, charge at pH 7.0, and molecular weight (including His_6 tag). (**B**) The numerical values for the apparent K_d of NTF2-YFP and NTF2W7AI64A-YFP in buffer, with 10% BSA, or 10% yeast lysate (YL) (graphically represented in Figure 3). Errors represent SEM (**C**) The numerical values for the apparent binding capacity of NTF2-YFP and NTF2W7AI64A-YFP buffer, with 10% BSA, or 10% YL (graphically represented in Figure 3). Errors represent SEM. (**D**) p-values for the indicated conditions were calculated using independent-samples two tailed student t-test.
